# IFN-γ Production Depends on IL-12 and IL-18 Combined Action and Mediates Host Resistance to Dengue Virus Infection in a Nitric Oxide-Dependent Manner

**DOI:** 10.1371/journal.pntd.0001449

**Published:** 2011-12-20

**Authors:** Caio T. Fagundes, Vivian V. Costa, Daniel Cisalpino, Flávio A. Amaral, Patrícia R. S. Souza, Rafael S. Souza, Bernhard Ryffel, Leda Q. Vieira, Tarcília A. Silva, Alena Atrasheuskaya, George Ignatyev, Lirlândia P. Sousa, Danielle G. Souza, Mauro M. Teixeira

**Affiliations:** 1 Immunopharmacology, Departamento de Bioquímica e Imunologia, Instituto de Ciências Biológicas, Universidade Federal de Minas Gerais, Belo Horizonte, Minas Gerais, Brazil; 2 Departmento de Microbiologia, Instituto de Ciências Biológicas, Universidade Federal de Minas Gerais, Belo Horizonte, Minas Gerais, Brazil; 3 Molecular Immunology and Embryology UMR6218, CNRS, Orleans, France; 4 Departamento de Bioquímica e Imunologia, Instituto de Ciências Biológicas, Universidade Federal de Minas Gerais, Belo Horizonte, Minas Gerais, Brazil; 5 Departmento de Patologia Oral, Faculdade de Odontologia, Universidade Federal de Minas Gerais, Belo Horizonte, Minas Gerais, Brazil; 6 Laboratory of Immunology Safety, State Research Center of Virology and Biotechnology “Vector”, Koltsovo, Novosibirsk Region, Russia; 7 State Institute of Standardizing and Control by Name of Tarasevich, Moscow, Moscow Province, Russia; 8 Departamento de Análises Clínicas e Toxicológicas, Faculdade de Farmácia, Universidade Federal de Minas Gerais, Belo Horizonte, Minas Gerais, Brazil; University of Rhode Island, United States of America

## Abstract

Dengue is a mosquito-borne disease caused by one of four serotypes of *Dengue virus* (DENV-1–4). Severe dengue infection in humans is characterized by thrombocytopenia, increased vascular permeability, hemorrhage and shock. However, there is little information about host response to DENV infection. Here, mechanisms accounting for IFN-γ production and effector function during dengue disease were investigated in a murine model of DENV-2 infection. IFN-γ expression was greatly increased after infection of mice and its production was preceded by increase in IL-12 and IL-18 levels. In IFN-γ^−/−^ mice, DENV-2-associated lethality, viral loads, thrombocytopenia, hemoconcentration, and liver injury were enhanced, when compared with wild type-infected mice. IL-12p40^−/−^ and IL-18^−/−^ infected-mice showed decreased IFN-γ production, which was accompanied by increased disease severity, higher viral loads and enhanced lethality. Blockade of IL-18 in infected IL-12p40^−/−^ mice resulted in complete inhibition of IFN-γ production, greater DENV-2 replication, and enhanced disease manifestation, resembling the response seen in DENV-2-infected IFN-γ^−/−^ mice. Reduced IFN-γ production was associated with diminished Nitric Oxide-synthase 2 (NOS2) expression and NOS2^−/−^ mice had elevated lethality, more severe disease evolution and increased viral load after DENV-2 infection. Therefore, IL-12/IL-18-induced IFN-γ production and consequent NOS2 induction are of major importance to host resistance against DENV infection.

## Author Summary

Dengue fever and its severe forms, dengue hemorrhagic fever and dengue shock syndrome, are the most prevalent mosquito-borne diseases on Earth. It is caused by one of four serotypes of *Dengue virus* (DENV-1–4). At present, there are no vaccines or specific therapies for dengue and treatment is supportive. Host response to infection is also poorly understood. Here, using a DENV-2 strain that causes a disease that resembles the severe manifestations of Dengue in humans, we demonstrate that IFN-γ production is essential for the host to deal with infection. We have also shown that IFN-γ production during DENV infection is controlled by the cytokines IL-12 and IL-18. Finally, we show that one of the mechanisms triggered by IFN-γ during host response to DENV infection is the production of Nitric Oxide, an important virustatic metabolite. Mice deficient for each of these molecules present marked increase in DENV replication after infection and more severe disease. Altogether, this study demonstrates that the IL-12/IL-18-IFN-γ-NO axis plays a major role in host ability to deal with primary DENV infection. These data bear relevance to the understanding of antiviral immune responses during Dengue disease and may aid in the rational design of vaccines against DENV infection.

## Introduction

Dengue fever (DF) and its severe forms, dengue hemorrhagic fever (DHF) and dengue shock syndrome (DSS), are mosquito-borne diseases caused by one of four serotypes of *Dengue virus* (DENV-1–4). Fifty to 100 million cases of DF are estimated annually mostly in tropical and subtropical regions of the world [1]–[3]. According to the World Health Organization (WHO), around 500,000 patients develop the severe forms of dengue and 20,000 deaths are estimated to occur each year. DHF is defined by the WHO as fever with hemorrhagic manifestations, thrombocytopenia, and hemoconcentration or other signs of plasma leakage [2]. Treatment of DF and of the severe forms of dengue infection is largely supportive. The large number of infected individuals, the lack of clinical or laboratory markers that indicate which patients will develop severe disease and the lack of specific treatment place an enormous burden on health systems of low income countries [2].

The pathogenesis of DENV infection remains poorly understood and involves a complex interplay of viral and host factors. Risk factors for severe disease include age [1], [4], viral serotype [1], [5] and genotype [1], [6], [7], and the genetic background of the host [1], [8], among others. Retrospective and prospective human studies have demonstrated that secondary infection by a heterologous serotype is the single greatest risk factor for DHF/DSS [9]–[11]. However, severe disease may also occur after primary infection [5], [12], [13]. In both cases, there appears to be a correlation between disease severity and viral load [9]–[13]. In addition, the immunopathogenesis of DENV probably involves the effects of cytokines on both infected and bystander immune cells, hepatocytes, and endothelial cells [2], [3], [13]. There are several studies which show enhanced levels of IFN-γ in dengue-infected humans but the precise role of IFN-γ in clinical dengue is somewhat controversial [14]–[16]. There are studies which suggest that levels of this cytokine may correlate positively with disease in humans [16], but other studies have shown that increased IFN-γ production correlated with higher survival rates in DHF patients [15]. In experimental systems, non-adapted viruses usually are unable to reach high viral loads, except in mice deficient for IFN receptors, suggesting that IFN-γ and its receptors are necessary for host resistance to Dengue infection [17]–[19]. However, the major cell types producing IFN-γ, mediators controlling production of this cytokine and major effector mechanisms triggered by IFN-γ are not known.

Optimal IFN-γ production in various infections models in mice is controlled by cytokines, especially IL-12 and IL-18 [20], [21]. The IFN-γ produced may then upregulate inducible nitric oxide synthase (NOS2), resulting in high levels of NO production by dendritic cells and macrophages [22]. NO is known to possess potent antiviral activities [22]. Therefore, in order to examine the role played by these molecules during dengue disease we conducted infection experiments in mice infected with an adapted strain of DENV-2. This unique DENV-2 strain was chosen because it was previously shown to induce in immunocompetent mice a disease that resembles severe dengue cases in humans [23]–[25], what does not happen with most non-adapted strains usually utilized in experimental settings [2], [3]. We show that disease is more severe and there are higher viral loads after DENV-2 infection of IFN-γ-deficient mice. Furthermore, we demonstrate that the combined action of IL-12 and IL-18 is necessary for optimal IFN-γ production and control of DENV-2 infection. Finally, we show that IFN-γ controls expression of NOS2 and NO production after DENV-2 infection and that NO production is crucial for resistance of the murine host to infection with DENV.

## Methods

### Ethics Statement

This study was carried out in strict accordance with the Brazilian Government's ethical and animal experiments regulations. The experimental protocol was approved by the Committee on the Ethics of Animal Experiments of the Universidade Federal de Minas Gerais (CETEA/UFMG, Permit Protocol Number 113/09). All surgery was performed under ketamine/xylazine anesthesia, and all efforts were made to minimize suffering. The guidelines followed by this Committee are based on the guidelines of Animal Welfare Act (AWA) and associated Animal Welfare Regulations (AWRs) and Public Health Service (PHS) Policy.

### Animals

Mice deficient for IFN-γ and NOS-2 were obtained from The Jackson Laboratory and were bred and maintained at the Gnotobiology and Immunology Laboratory of Instituto de Ciências Biológicas. Mice deficient for IL-12p40 were kindly provided by Dr. J. Magran through Dr. L. V. Rizzo (Instituto de Ciências Biomédicas (ICB), University of São Paulo, São Paulo, Brazil) and were bred and maintained at the Gnotobiology and Immunology Laboratory of Instituto de Ciências Biológicas. Mice deficient for IL-18 [26] were kindly provided by Dr. F.Q. Cunha and were bred and maintained at the Gnotobiology and Immunology Laboratory of Instituto de Ciências Biológicas. Mice deficient for IL-23p19 [27] were bred and maintained at the animal facility of the Transgenose Institute (CNRS, Orleans). All mice were on C57BL/6J genetic background (back-crossed at least 10 times) and wild-type control C57BL/6J (WT) mice were used, except for IL-18-deficient mice, that were on the BALB/c background and were compared to their proper WT littermates. For experiments, 7–10 weeks old mice were kept under specific pathogen–free conditions, in filtered-cages with autoclaved food and water available *ad libitum*.

### Virus

An adapted Dengue virus 2 (DENV-2) strain was obtained from the State Collection of Viruses, Moscow, Russia [23]. Briefly, the virus had undergone two passages in the brain of BALB/c suckling mice. Five days after infection, brains of moribund mice were harvested for preparing 10% (w/v) brain suspension in modified Eagle's medium (MEM). After that, eight sequential passages through BALB/c mice of different ages (1–4 weeks old) by intraperitoneal (i.p.) injection were performed. Two sequential passages were carried out for each age group of. After each passage, the brains of the moribund mice were harvested for preparing 10% brain suspension and then used for the next passage. The last passage of DENV-2 strain P23085 was performed in neonatal mice to produce stocks which were stored as 10% brain suspension at −70°C. Sequences of portions of E and NS1 genes of the adapted virus were deposited previously at GenBank under the accession number AY927231 [22]. Virus adaptation was performed in a biosafety level-3 (BSL-3) facility of the SRC VB «Vector», Russia, Koltsovo. After adaptation, monolayers of *Aedes albopictus* C6/36 cell line were infected with DENV-2 strain P23085 at a multiplicity of infection (MOI) of 0.05 PFU/cell and incubated at 28°C for 5–7 days. The cultured medium was harvested after cytopathic effect was noticed and cell debris removed by centrifugation. The virus supernatant was collected and stored at −70°C until use. The cultured medium of mock-infected monolayers of *Aedes albopictus* C6/36 cell line was used as control of the infection. To calculate virus titer, expressed as LD_50_, in the harvested cultured medium, groups of ten mice were inoculated i.p. with serial dilutions of the virus and lethality recorded. The titer of our DENV-2 stock was 10^5^ LD_50_/ml of suspension, as calculated in 8–10-week-old BALB/c mice. 1LD_50_ corresponded to 20 PFU of the adapted DENV-2 strain.

### Experimental procedure

For infection experiments, the virus-containing cell-supernatant was diluted in endotoxin-free PBS and injected i.p. into mice. For the evaluation of lethality, mice were inoculated i.p. with DENV-2 virus and lethality rates evaluated every 12 h. The various other parameters were evaluated at 3, 5 or 7 days after i.p. inoculation of the virus. In all experiments using genetically deficient mice, experiments with the relevant WT controls were performed in parallel. Non-infected (NI) animals were inoculated with suspension from non-infected cell supernatant diluted in a similar manner. In the experiments involving genetically deficient mice, the NI group represents the pooled results obtained from the analysis of deficient mice and WT non-infected mice. Results were pooled for ease of presentation.

In some experiments IL-18 was neutralized by daily i.p. injection of 250 µg of recombinant human IL-18BP per animal (hIL-18 bp), starting 1 hour after DENV-2 inoculation until day 4 after virus inoculation. The dose was chosen based in a previous study [28]. Control animals received PBS. The hIL-18 bp isoform was a kind gift of Dr. Amanda Proudfoot from Merck-Serono Pharmaceuticals (Geneve, Switzerland).

### Cell culture and in vitro infection studies

Murine bone marrow cells were isolated from femurs and were differentiated into myeloid DCs after culturing at 2×10^6^ cells/ml for 10 days in RPMI supplemented with 10% FBS and 4% J558L cell-conditioned medium as a source of GM-CSF as described [29]. DCs were plated in 96-well microculture plates (at 2×10^5^ cells/well in DMEM supplemented with 2 mM l-glutamine and 2×10^−5^ M 2-ME) and for infection, cells were incubated with 50 µL of the cell supernatant suspension containing DENV-2 at 0,01 MOI in the presence or not of recombinant murine IFN-γ (100 U/ml). Negative controls were stimulated with sterile cell supernatant obtained from mock infected cells.

### Titration of virus

Mice were assayed for viral titers in spleen. For virus recovery in spleen, the organ was collected aseptically and stored at −70°C until assayed for DENV-2 virus. Tissue samples were weighed, grounded by using a pestle and mortar and prepared as 10% (w/v) homogenates in minimal essential medium (MEM) without fetal bovine serum (FBS). Viral load in the supernatants of tissue homogenates assessed by direct plaque assays using LLC-MK2 cells cultured in agarose overlay. Briefly, organ homogenates were diluted serially and a 0.4 ml volume placed in duplicate into each of 6-wells of LLC-MK2 cell monolayers and incubated for 1 h. An overlay solution containing 2× MEM and 1% agarose in equal volumes was added to each well and the cultures incubated for 7 days. Cultures were stained with crystal violet for enumeration of viral plaques. Cell monolayers incubated with tissue homogenates of not infected mice were used as control for the assay. The results were measured as plaque forming units (PFU) per gram of tissue weight. The limit of detection of the assay was 100 PFU/g of tissue.

### Measurement of cytokine/chemokine concentrations

The concentration of cytokines (TNF-α, IFN-γ, IL-6, IL-12p40, IL-12p70 and IL-18) in serum or tissue samples was measured using commercially available antibodies and according to the procedures supplied by the manufacturer (R&D Systems, Minneapolis, except for IL-18, manufactured by BD Pharmingen). Serum was obtained from coagulated blood (15 min at 37°, then 30 min a 4°C) and stored at −20°C until further analysis. One hundred milligrams of tissues (liver and spleen) was homogenized in 1 ml of PBS containing anti-proteases (0.1 mM phenylmethilsulfonyl fluoride, 0.1 mM benzethonium chloride, 10 mM EDTA and 20 KI aprotinin A) and 0.05% Tween 20. The samples were then centrifuged for 10 min at 3000 *g* and the supernatant immediately used for ELISA assays. The detection limit of the ELISA assays was in the range of 4–8 pg/ml.

### Quantification of nitrite in cell supernatants

Cell-free culture medium was obtained by centrifugation and assayed for nitrite content, determined by the Griess method [30]. For this assay, 0.1 ml of culture medium was mixed with 0.1 ml of Griess reagent in a multiwell plate, and the absorbance at 550 nm read 10 min later. The NO_2_^−^ concentration (µM) was determined by reference to a NaNO_2_ standard curve.

### Evaluation of blood parameters

Blood was obtained from the brachial plexus in heparin-containing syringes at the indicated times. The final concentration of heparin was 50 u/ml. Platelets were counted in a Coulter Counter (S-Plus Jr). Results are presented as number of platelets per µl of blood. For the determination of the hematocrit, a sample of blood was collected into heparinized capillary tubes and centrifuged for 10 min in a Hematocrit centrifuge (HT, São Paulo, Brazil).

### Transaminase activity

Aspartate transaminase activity was measured in individual serum samples, using a commercially available kit (Bioclin, Belo Horizonte, Brazil). Results are expressed as the U/dL of serum.

### Real Time PCR

Total RNA was isolated from Spleen of mice for evaluation of NOS2 mRNA expression. RNA isolation was performed using Illustra RNAspin Mini RNA Isolation Kit (GE Healthcare). The RNA obtained was resuspended in diethyl pyrocarbonate treated water and stocked at −70°C until use. Real-time RT-PCR was performed on an ABI PRISM 7900 sequence-detection system (Applied Biosystems) by using SYBR Green PCR Master Mix (Applied Biosystems) after a reverse transcription reaction of 2 µg of total RNA by using M-MLV reverse transcriptase (Promega). The relative level of gene expression was determined by the comparative threshold cycle method as described by the manufacturer, whereby data for each sample were normalized to hypoxanthine phosphoribosyltransferase and expressed as a fold change compared with non-infected controls. The following primer pairs were used: *hypoxanthine phosphoribosyltransferase*, 5′-GTTGGTTACAGGCCAGACTTTGTTG-3′ (forward) and 5′-GAGGGTAGGCTGGCCTATAGGCT-3′ (reverse); and *nos2*, 5′- CCAAGCCCTCACCTACTTCC -3′ (forward) and 5′- CTCTGAGGGCTGACACAAGG -3′ (reverse).

### FACS analysis

Spleen cells were evaluated *ex vivo* for extracellular molecular expression patterns and for intracellular cytokine expression patterns. Briefly, spleens were removed from infected mice at the indicated timepoints. Then cells were isolated, and immediately stained for surface markers, fixed with 2% formaldehyde and then permeabilized with a solution of saponin and stained for 30 min at room temperature, using conjugated anti-IFN-γ monoclonal antibodies. Preparations were then analyzed using a FACScan (Becton Dickinson), and 50 000 gated events on total lymphocyte/monocyte population were acquired for later analysis. Figure S1A shows the gating strategy utilized for IFN-γ^+^ population analysis in CD4^+^ cells. Briefly, lymphocyte/monocyte population was isolated in gate R1. At this region, the cell population positive for the surface marker of interest was isolated (R2) and among cells in this region, IFN-γ^+^ cells were obtained (R3). Analogous strategies were utilized for the other several populations studied. The antibodies used for the staining were rat immunoglobulin controls, anti-CD4-PE, anti-CD8-PE, anti-NK1.1-PE, anti-CD3- PE-Cy5 and anti-IFN-γ-FITC (all from Biolegend Inc). Analysis was conducted using the software Flow Jo 7.2 (Tree Star Inc).

### Histopathology and immunohistochemestry

A portion of liver was obtained from killed mice at the indicated time points, immediately fixed in 10% buffered formalin for 24 hours and tissues fragments were embedded in paraffin. Tissue sections (4 µm thick) were stained with hematoxylin and eosin (H&E) and examined under light microscopy or collected in serial sections on glass slides coated with 2% 3-aminopropyltriethylsilane (Sigma Aldrich, St. Louis, MO). The latter sections were deparaffinized by immersion in xylene, and this was followed by immersion in alcohol and then incubation with 3% hydrogen peroxide diluted in Tris-buffered saline (TBS) (pH 7.4) for 30 minutes. The sections were then immersed in citrate buffer (pH 6.0) for 20 minutes at 95°C for antigen retrieval. The slides were then incubated with the rabbit polyclonal anti-NOS2 (N-20, sc-651, Santa Cruz Biotechnology, Santa Cruz, CA) diluted 1∶100; at 4°C overnight in a humidified chamber. After washing in TBS, the sections were treated with a labeled streptavidin-biotin kit (LSAB, K0492, Dako, Carpinteria, CA). The sections were then incubated in 3,3′-Diaminobenzidine (K3468, Dako) for 2 to 5 minutes, stained with Mayer's hematoxylin and covered. Negative controls were obtained by the omission of primary antibodies, which were substituted by 1% PBS-BSA.

### Statistical analysis

Results are shown as means ± SEM. Differences were compared by using analysis of variance (ANOVA) followed by Student-Newman-Keuls post-hoc analysis. Differences between lethality curves were calculated using Log rank test (Graph Prism Software 4.0). Results with a P<0.05 were considered significant.

## Results

### IFN-γ production is necessary for host resistance to DENV primary infection

**Figure 1:**
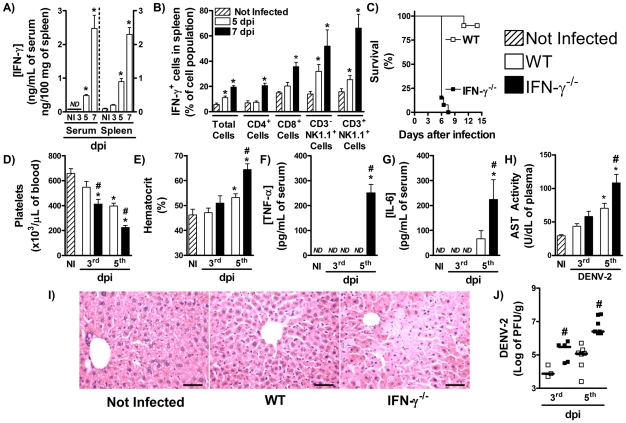
IFN-γ-deficient mice are highly susceptible to DENV infection. (A) WT mice were inoculated with 10LD_50_ of DENV-2 and at the indicated timepoints, the following parameters were assessed: IFN-γ concentration in serum (left panel) and spleen (right panel), measured by ELISA (A); IFN-γ intracellular staining in splenic cells, assessed by FACS analysis (B). (C–J) WT and IFN-γ^−/−^ mice were inoculated with 10LD_50_ of DENV-2 and at the indicated timepoints, the following parameters were assessed: lethality rates after infection (C); platelet counts (D) and hematocrit (E) in blood; TNF-α (F) and IL-6 (G) concentration, measured by ELISA, and AST activity (H), measured by colorimetric assay, in serum; Liver injury, assessed by Hematoxylin & Eosin staining (five days after infection) (I); Viral loads recovered from the spleen, by plaque assay (J). Results are expressed as mean ± SEM (except for J, expressed as median) and are representative of at least two independent experiments. N = 5 mice per group * P<0.05 *vs.* NI. # P<0.05 *vs.* WT. NI: Not infected. ND: Not detected. dpi:day post-infection.

An initial set of experiments were carried out to assess the kinetics of IFN-γ production and major IFN-γ producing cell types after DENV-2 infection. As shown in Figure 1, there was an increase in serum and splenic levels of IFN-γ from the 5^th^ day of infection (Figure 1A). Levels of IFN-γ enhanced further at day 7 in both serum and spleen (Figure 1A). In spleen, IFN-γ staining was detected in about 10% of total cells in the 5^th^ day after inoculation and reached about 15% at the 7^th^ day post infection (Figure 1B and Figure S1B). CD3^−^NK1.1^+^ NK cells and CD3^+^NK1.1^+^ NKT populations presented increased proportions of IFN-γ staining at the 5^th^ day post infection (Figure 1B and Figure S1E and S1F). In addition, there was increase in expression of IFN-γ on all cell populations analyzed at day 7 after infection (Figure 1B). Significantly, over 30% of CD4^+^ T cells, 25% of CD8^+^ T cells, 40% of CD3^−^NK1.1^+^ NK cells and CD3^+^NK1.1^+^ NKT cells were IFN-γ^+^ at day 7 after infection (Figure 1B and Figures S1C–F). When the gate was set at IFN-γ^+^ cells, the majority of IFN-γ^+^ cells were CD8^+^ T cells (30±3%) and CD4^+^ T cells (25±1%).

To investigate the role played by IFN-γ during DENV infection, WT and IFN-γ-deficient (IFN-γ^−/−^) mice were inoculated DENV-2 and lethality rates and disease course evaluated. As seen in Figure 1C, 100% of IFN-γ^−/−^ mice were dead before the seventh day of infection, and only 15% of WT mice had succumbed to infection. This early lethality of IFN-γ^−/−^ mice was characterized by more severe manifestation of disease after DENV infection. Three days after infection, IFN-γ^−/−^ mice already presented reduced platelets counts (Figure 1D), and at the 5^th^ day of infection, there was marked thrombocytopenia (Figure 1D) and significant increase in hematocrit values (Figure 1E) in IFN-γ^−/−^ mice when compared to WT mice. In addition to the alterations seen in hematological parameters, there was enhanced production of pro-inflammatory cytokines after infection. As shown in Figures 1F and 1G, there were no detectable levels of TNF-α and IL-6 in serum of WT mice at day 5 after DENV-2 infection. However, both cytokines were significantly elevated in serum of infected IFN-γ^−/−^ mice (Figures 1F and 1G). Infected-IFN-γ^−/−^ mice showed hepatic injury, as assessed by increased AST activity in plasma of IFN-γ^−/−^ mice in the 5^th^ day of infection (Figure 1H). There was also marked changes in liver architecture. WT mice inoculated with DENV-2 had little changes in liver, as assessed by histology. In contrast, there were signs of congestion and hepatocyte degeneration and necrosis in infected IFN-γ^−/−^ mice (Figure 1I). In addition to the greater disease severity observed, IFN-γ^−/−^ mice presented greater viral replication after infection than in WT mice. At the 3^rd^ day of infection, IFN-γ^−/−^ mice presented a 10 fold increase in DENV-2 viral loads in spleen and DENV-2 titers in spleen of infected-IFN-γ^−/−^ mice were above 1.5 log greater than in infected-WT mice in the 5^th^ day of infection (Figure 1J). Therefore, the data depicted here show IFN-γ is expressed and plays an important role in host defense against DENV infection.

### IL-12 and IL-18 control IFN-γ production during DENV infection

**Figure 2:**
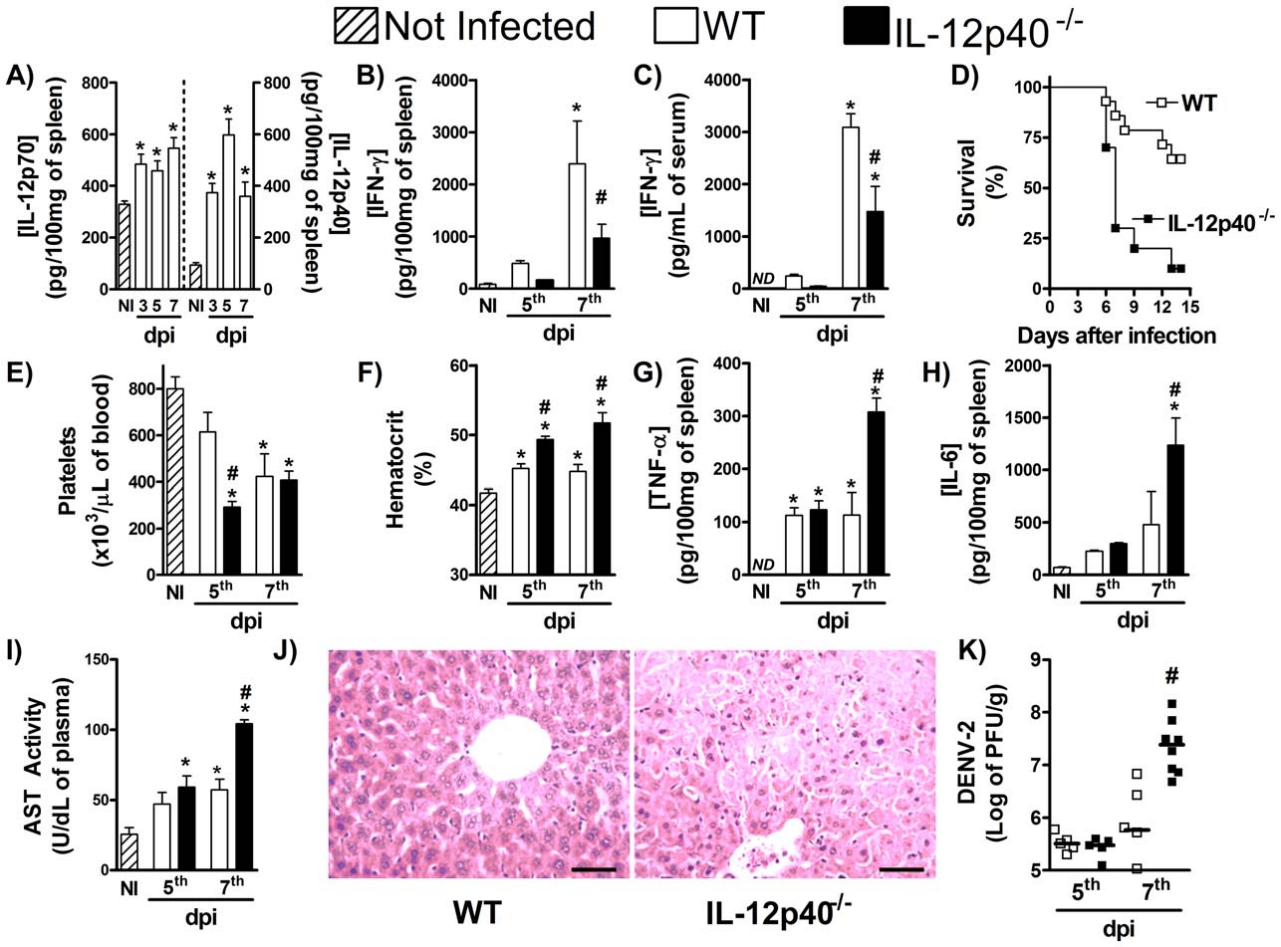
IL-12 controls production of IFN-γ and host resistance to DENV infection. (A) WT mice were inoculated with 10LD_50_ of DENV-2 and at the indicated timepoints, IL-12p70 (left panel) and IL-12p40 (right panel) concentration in spleen were determined by ELISA. (B–K) WT and IL-12p40^−/−^ mice were inoculated with 10LD_50_ of DENV-2 and at the indicated timepoints, the following parameters were assessed: IFN-γ concentration in spleen (B) and serum (C), measured by ELISA; lethality rates after infection (D); platelet counts (E) and hematocrit (F) in blood; TNF-α (G) and IL-6 (H) concentration, measured by ELISA, and AST activity (I), measured by colorimetric assay, in serum; Liver injury, assessed by Hematoxylin & Eosin staining (seven days after infection) (J); Viral loads recovered from the spleen, by plaque assay (K). Results are expressed as mean ± SEM (except for J, expressed as median) and are representative of at least two independent experiments. N = 6 mice per group. * P<0.05 *vs.* NI. # P<0.05 *vs.* WT. NI: Not infected. ND: Not detected. dpi:day post-infection.

Our next objective was to evaluate the roles of IL-12 and IL-18 in controlling IFN-γ production by the murine host during DENV infection. After DENV-2 infection, there were detectable levels of both IL-12p70 and IL-12p40 in the spleen of WT mice already in the 3^rd^ day of infection (Figure 2A). The concentration of both cytokines was increased in the 5^th^ and remained above background levels at the 7^th^ day of infection (Figure 2A). This early production is consistent with a putative role of IL-12 in inducing IFN-γ production. Consistently with the latter possibility, there was a drastic reduction in IFN-γ production after DENV-2 infection of IL-12p40^−/−^ mice, which are deficient for both IL-12 and IL-23 production (Figures 2B and 2C). In keeping with the relevance of IFN-γ during dengue infection and reduced IFN-γ production, there was enhanced lethality rates (Figure 2D), increased thrombocytopenia (Figure 2E) and enhanced hemoconcentration (Figure 2F) after DENV-2 infection of IL-12p40^−/−^ mice. There were higher concentrations of TNF-α (Figure 2G) and IL-6 (Figure 2H) in spleen and more severe hepatic injury in IL-12p40^−/−^ than WT mice after infection (Figure 2I and 2J). Finally, IL-12p40 deficiency resulted in greater loads of DENV-2 in spleen at the 7^th^ day after infection, when compared with WT-infected mice (Figure 2K). The reduction of IFN-γ production and the more severe disease seen in IL-12p40^−/−^ mice seem to be specifically due to IL-12 deficiency as IL-23p19^−/−^-deficient mice produced similar amounts of IFN-γ after DENV-2 infection (Supplementary Figure S2A) and presented a disease of similar intensity (Figure S2B and S2C) and unaltered viral loads (Figure S2 D) when compared to infected-WT mice.

**Figure 3:**
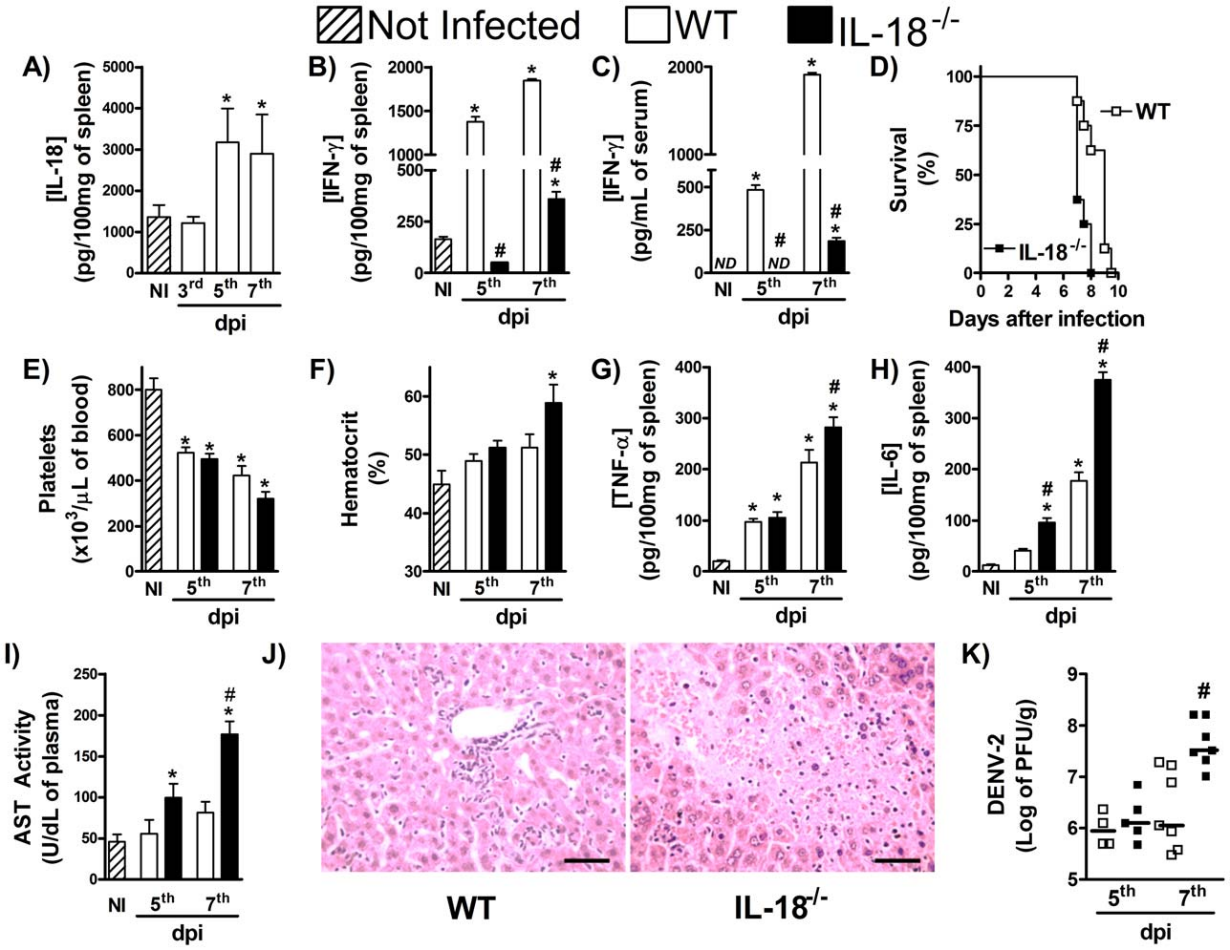
IL-18 controls production of IFN-γ and host resistance to DENV infection. (A) WT mice were inoculated with 10LD_50_ of DENV-2 and at the indicated timepoints, IL-18 concentration in liver were determined by ELISA. (B–K) WT and IL-18^−/−^ mice were inoculated with 10LD_50_ of DENV-2 and at the indicated timepoints, the following parameters were assessed: IFN-γ concentration in spleen (B) and serum (C), measured by ELISA; lethality rates after infection (D); platelet counts (E) and hematocrit (F) in blood; TNF-α (G) and IL-6 (H) concentration in spleen, measured by ELISA; AST activity in serum (I), measured by colorimetric assay; Liver injury, assessed by Hematoxylin & Eosin staining (seven days after infection) (J); Viral loads recovered from the spleen, by plaque assay (K). Results are expressed as mean ± SEM (except for J, expressed as median) and are representative of at least two independent experiments. N = 6 mice per group. * P<0.05 *vs.* NI. # P<0.05 *vs.* WT. NI: Not infected. ND: Not detected. dpi:day post-infection.

Another cytokine shown to induce IFN-γ production during infections is IL-18 [21]. In the present study, IL-18 concentrations rose rapidly in liver at the 3^rd^ day of DENV-2 infection, but returned to basal levels in the subsequent timepoints evaluated (Figure 3A). There was marked reduction of IFN-γ production in spleen and serum of DENV-2-infected IL-18^−/−^ mice when compared with WT infected mice (Figure 3B and 3C, respectively). Available IL-18^−/−^ mice were in the BALB/c background which we have previously shown to be more susceptible to DENV2-induced disease and lethality [24]. Indeed, all WT mice in the BALB/c background were dead by day 10 of DENV-2 infection using an inoculum that caused little lethality in C57Bl/6 mice (compare Figures 3D and 1C). All IL-18^−/−^ mice also succumbed to infection but mice died earlier than WT controls after DENV-2 infection (p = 0.0237) (Figure 3D). Although the degree of thrombocytopenia was similar in both strains of mice (Figure 3E), hemoconcentration was greater in IL-18^−/−^ than WT infected mice (Figure 3F). Levels of TNF-α (Figure 3G) and IL-6 (Figure 3H) and severity of liver injury (Figure 3I and 3J) occurred to a greater extent in spleens of IL-18^−/−^ than WT infected mice (Figure 3G and 3H). Significantly, enhanced clinical disease and earlier deaths were accompanied by elevation in viral loads in spleen of IL-18^−/−^ mice (Figures 3K).

**Figure 4:**
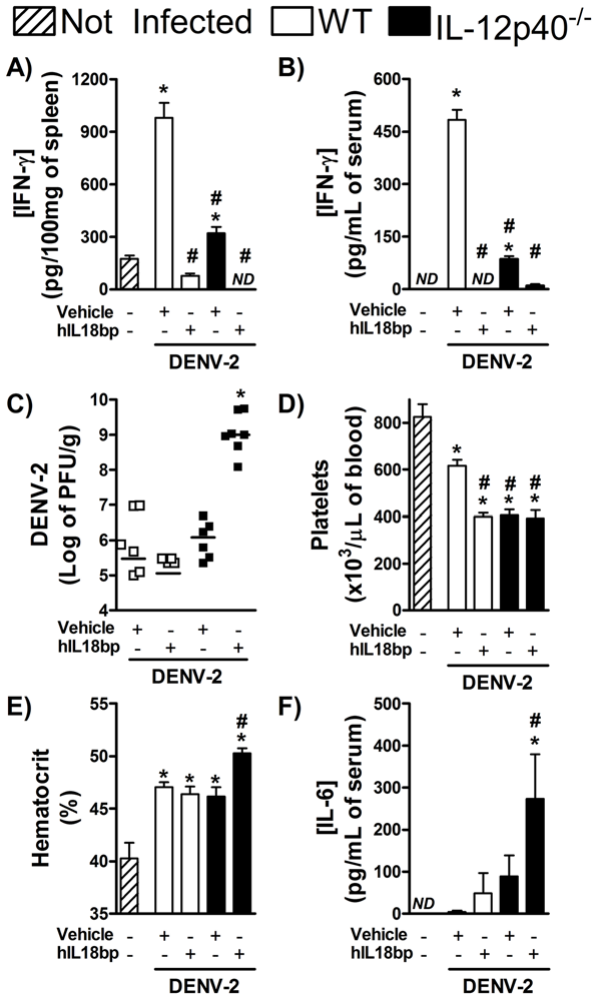
IL-12 and IL-18 act in synergism to induce IFN-γ production and resistance to DENV infection. WT and IL-12p40^−/−^ mice (*n* = 5 mice per group), treated or not with IL-18 bp (daily i.p. injection of 250 µg of protein), were inoculated with 10LD_50_ of DENV-2 (i.p) and, 5 days after infection, The following parameters were assessed: IFN-γ concentration in spleen (A) and serum (B) , measured by ELISA; viral loads recovered from the spleen, measured by plaque assay (C); platelets counts (D) and hematocrit (E) in blood; IL-6 concentration in serum, measured by ELISA (F); Results are expressed as mean ± SEM (except for A, expressed as median) and are representative of at least two independent experiments. N = 5 mice per group. * P<0.05 *vs.* NI. # P<0.05 *vs.* WT. NI: Not infected. ND: Not detected.

The phenotype of either IL-12^−/−^ or IL-18^−/−^ mice were not as severe as the phenotype of IFN-γ^−/−^ mice. For example, whereas viral loads were already approximately 2 log greater at day 5 in IFN-γ^−/−^ mice, this was not the case in IL-12^−/−^ or IL-18^−/−^ mice (Figures 2J and 3J). Indeed, IFN-γ production was not abolished in IL-12^−/−^ or IL-18^−/−^ mice and viral loads were only significantly different from WT at day 7 after infection (see Figures 2J and 3J). In order to block simultaneously the action of both IL-12 and IL-18, IL-12p40^−/−^ mice were treated with IL-18 bp at doses shown to block IL-18 action [28]. Treatment of IL-12p40^−/−^ mice with IL-18 bp also resulted in total abrogation of IFN-γ levels in serum (Figure 4A) or spleen (Figure 4B) of infected mice. Treatment of IL-12p40^−/−^ with IL-18 bp also resulted in marked enhancement of viremia already at day 5 after infection (Figure 4C), results which are similar to those obtained in IFN-γ^−/−^ mice (Figure 1I) and substantially different from results observed at day 5 in IL-12p40^−/−^ mice or mice treated with IL-18 bp alone (Figure 4C). Moreover, treatment of IL-12p40^−/−^ with IL-18 bp resulted in thrombocytopenia, which was similar to that observed in IL-12p40^−/−^ or IL-18 bp-treated mice (Figure 4D), and hemoconcentration, which was greater than in the other groups (Figure 4E). Levels of IL-6 in plasma were also further enhanced by the treatment of IL-12p40^−/−^ mice with IL-18 bp than in either condition alone (Figure 4F). The enhanced viral load and greater disease severity already at day 5 resulted in greater lethality rates in IL-12p40^−/−^ mice treated with IL-18 bp than in either condition alone or WT mice (Lethality rate at day 7: WT mice, 0%; IL-18bp-treated mice, 0%; IL-12p40^−/−^ mice, 33%; IL-12p40^−/−^ mice+IL-18 bp, 83%, n = 6). In concert, the data presented above suggest that IL-12 and IL-18 act together to induce optimal IFN-γ production during dengue infection in mice.

### IFN-γ-mediated protection to DENV infection involves elevation of NOS2-mediated NO production

**Figure 5:**
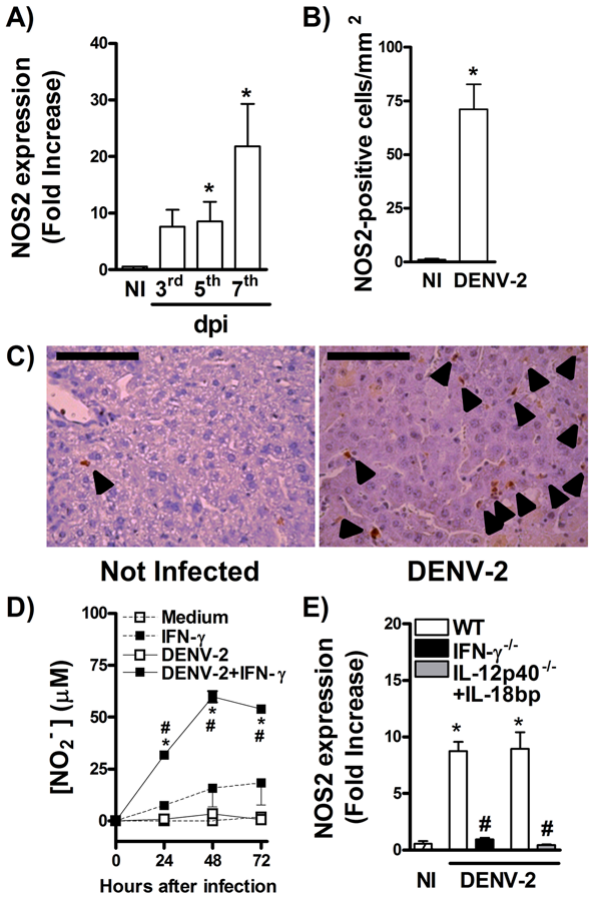
IFN-γ controls NOS2-mediated NO production during DENV infection. (A–C) WT mice were inoculated with 10LD_50_ of DENV-2 and at the indicated timepoints, the following parameters were assessed: NOS2 RNA expression in spleen, determined by qPCR (A); NOS2 staining in liver, assessed by IHC at the 7^th^ day of infection (B, C). (D) Bone marrow derived dendritic cells were infected with DENV-2 (MOI 0,1 PFU/cell) in the presence or not of IFN-γ, and at the indicated timepoints, NO production was quantified by Griess reaction. (E). WT, IFN-γ^−/−^ and hIL-18 bp-treated IL-12p40^−/−^ mice (daily i.p. injection of 250 µg of protein, n = 5 mice per group) were inoculated with 10LD_50_ of DENV-2 (i.p) and in the fifth day of infection NOS2 RNA expression was determined by qPCR. Results are shown as fold increase over basal expression in control mice (A, E); number of positive cells per mm^2^ of liver (B); and µM of nitrite in medium (D). Results are expressed as mean ± SEM and are representative of at least two independent experiments. N = 5 mice per group. * P<0.05 *vs.* NI. # P<0.05 *vs.* WT. In (E), * P<0.05 *vs.* DENV-2 infected cell, and # for P<0.05 *vs.* medium or IFN-γ-treated cells. dpi:day post-infection.

Nitric Oxide production by phagocytes is a well known effector mechanism induced by IFN-γ during host response to infections [22]. To assess whether this pathway is relevant in host response to DENV infection, we evaluated NOS2 expression after DENV-2 infection. As shown in Figure 5A, there was increase in NOS2 mRNA expression in spleen already at day 5 day but expression rose rapidly at day 7 after DENV2 infection of WT mice (Figure 5A). Evaluation of NOS2 staining in the liver by immunohistochemistry showed significant NOS2 expression, virtually only in infiltrating leukocytes, at day 7 after infection (Figure 5B, C). Consistently with the ability of IFN-γ to induce NOS2, there was no production of NO by dendritic cells infected with DENV-2, *in vitro* (Figures 5D). However, treatment of dendritic cells with IFN-γ prior to infection resulted in production of significant amounts of NO (Figure 5D). In addition, expression of NOS2 was greatly decreased in spleen of IFN-γ^−/−^ mice after DENV-2 infection (Figure 5E). As IL-12 and IL-18 cooperate for optimal induction of IFN-γ (results above), we evaluated whether treatment of IL-12p40^−/−^ mice with IL-18 bp would also results in reduced NOS2 expression in spleen. As seen in Figure 5E, concomitant absence of both IL-12 and IL-18 led to impaired NOS2 expression in spleen that was quantitatively similar to results obtained in IFN-γ^−/−^ mice (Figure 5E).

**Figure 6:**
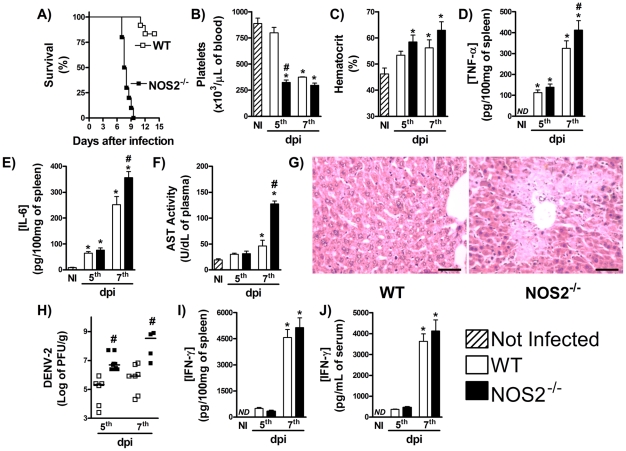
NOS2-deficient mice are more susceptible to DENV infection. WT and NOS2^−/−^ mice were inoculated with 10LD_50_ of DENV-2 and at the indicated timepoints, the following parameters were assessed: lethality rates after infection (A); platelet counts (B) and hematocrit (C) in blood; TNF-α (D) and IL-6 (E) concentration in spleen, measured by ELISA; AST activity in serum (F), measured by colorimetric assay; Liver injury, assessed by Hematoxylin & Eosin staining (seven days after infection) (G); Viral loads recovered from the spleen, by plaque assay (H). IFN-γ concentration in spleen (I) and serum (J) measured by ELISA; Results are expressed as mean ± SEM (except for H, expressed as median) and are representative of at least two independent experiments. N = 6 mice per group. * P<0.05 *vs.* NI. # P<0.05 *vs.* WT. NI: Not infected. ND: Not detected. dpi:day post-infection.

To assess the role played by NOS2-induced NO during DENV infection, NOS2^−/−^ mice were inoculated with DENV-2 and lethality rates and hematological alterations monitored. As shown in Figure 6A, NOS2^−/−^ mice were markedly susceptible to DENV infection, as all knockout animals but none of WT mice were dead by the 10^th^ day of infection. Thrombocytopenia (Figures 6B) was more intense earlier but hemoconcentration was similar in both groups (Figure 6C). There was enhanced splenic production of TNF-α (Figure 6D) and IL-6 (Figure 6E) and greater hepatic injury (Figure 6F and 6G) after DENV-2 infection of NOS2^−/−^ than WT mice. Importantly, viral loads in spleen after DENV-2 infection were significantly greater in NOS2^−/−^ than WT mice (Figures 6H). Of note, all alterations seen in NOS2^−/−^-infected mice were not due to reduction in IFN-γ production after infection. Indeed, IFN-γ levels in spleen and serum were similar in WT and NOS2^−/−^ infected mice (Figures 6I and 6J). Therefore, NOS2-derived NO production is driven by IFN-γ and is essential for host protection during DENV primary infection.

## Discussion

The major findings of the present study can be summarized as follows: 1) IFN-γ production is essential for host resistance to DENV infection. NK and NKT cells are the sources of IFN-γ during the early periods of infection and are followed by CD4^+^ and CD8^+^ T cells, which are the main producers at the peak of host response to infection; 2) production of IL-12 and IL-18 precedes IFN-γ and optimal IFN-γ production relies on the combined action of IL-12 and IL-18; and 3) IFN-γ is essential for NOS2 induction and NOS2 plays an important role in controlling virus replication. These studies, therefore, indicate that IL-12/IL-18-induced IFN-γ production and consequent induction of NOS2 are essential for murine host response to DENV infection.

Previous studies support a protective role played by IFN-γ during host response to DENV infection. For example, Shresta and coworkers have shown that IFN-γ receptor-deficient mice were more susceptible to DENV-induced lethality than WT-infected mice, despite no differences in viral loads in several target organs between both groups [17]. The increased susceptibility was enhanced further when type I IFN receptor was also absent, and deficiency in both cytokine receptors resulted in disseminated viral replication [17]. In this respect, IFN receptors-deficient mice (AG129 strain) are known to be permissive for replication of DENV clinical isolates in peripheral tissues and CNS, and represent a well established experimental model of DENV infection [17]–[19]. In the present work, we have demonstrated that IFN-γ is produced as early as the fifth day of infection in WT mice and lack of IFN-γ action culminated in early lethality to a sublethal inoculum. These data establish IFN-γ as essential for host control of DENV replication and resistance to infection. The correlation between increased IFN-γ production and higher survival rates in DHF patients [15] also supports this idea.

Of note, enhanced viral replication in IFN-γ-deficient mice was associated with more severe disease manifestation, as showed by enhanced hematological alterations and hepatic injury. More severe disease was also noticed in DENV-infected AG129 mice, characterized by paralysis and elevated hematocrit [17]. Importantly, Gunther and colleagues have demonstrated in a human challenge model of DENV infection that only sustained IFN-γ production was associated with protection against fever and viremia during the acute phase of illness [31]. These data suggest that IFN-γ is important to prevent worsening of disease. In humans, epidemiological studies have shown that a substantial number of patients with severe disease have evidence of a previous infection with a distinct serotype [1]–[3], [9]–[11], [32]. Several hypotheses have been raised to explain this immune-mediated enhancement of disease severity. For example, it has been hypothesized that subneutralizing levels of antibodies facilitate the entry of viral particles in permissive cells (a phenomenon termed antibody-dependent enhancement - ADE), enhancing viral load, and exacerbating disease manifestation [33]. Experimental DENV models support this hypothesis and suggest that disease severity is directly associated with enhanced viral replication during infection [34], [35]. Of note, infected IFN-γ-deficient mice, as well as IL-12p40^−/−^ and IL-18^−/−^ infected mice, presented elevated viral loads, in parallel with elevated hematocrits, thrombocytopenia, and liver injury. Therefore, we may suggest that the worse outcome seen in mice with reduced IFN-γ production after infection is due to inability in control of DENV replication, leading to viral burden and enhancement of disease.

Mice in which IFN-γ production was decreased or deficient had a significant increase in levels of pro-inflammatory mediators after DENV infection. Indeed, both TNF-α and IL-6 production were enhanced in DENV-2 infected IFN-γ^−/−^, IL-12p40^−/−^, and IL-18^−/−^ mice, when compared with WT controls. Increased levels of these cytokines have been associated with severity of dengue manifestation in humans [36]–[38]. Hence, enhanced TNF-α release by T cells during secondary stimulation with DENV antigens was found in hospitalized patients with more severe disease evolution [39]. In addition, the ratio of TNF-α-producing to IFN-γ-producing T cells among peripheral blood mononuclear cells from dengue-vaccine recipients was shown to be greater after in vitro stimulation with antigen from heterologous dengue serotypes [39], suggesting that increased amounts of TNF-α alters response to infection and may result in more-severe disease manifestation. Findings in murine experimental models support this idea [40]. Altogether, these findings in humans suggest that IFN-γ production is associated with protective responses to DENV infection and that severe disease may occur due to absence of proper IFN-γ release and to enhanced TNF-α production during response, although it remains to be shown if enhanced TNF-α production seen in DENV infected IFN-γ^−/−^ mice was due to T cells or to any other cellular population.

Interestingly, enhanced viral load have also been associated with increased pro-inflammatory response during mouse experimental infection by West Nile virus [41], another important flavivirus that is pathogenic to humans. The latter findings support the hypothesis that increased virus replication in the absence of IFN-γ production leads to increased pro-inflammatory mediators response. TNF-α blockade in experimental models of DENV infection resulted in prevention of disease [19], [23] and TNF-α action has been implicated in increased vascular permeability after infection in experimental settings [13]. Of note, inhibition of other pro-inflammatory mediators produced in the evaluated experimental model of DENV infection, including PAF and MIF, is associated with reduced production of TNF-α and IL-6 and milder disease manifestation, reduced hypotension and vascular permeability after DENV infection [13], [24], [25]. Hepatic injury was also enhanced in IFN-γ^−/−^ mice infected with DENV. Data from our laboratory suggest that enhanced liver injury during experimental DENV infection involves both productive viral infection of hepatocytes and immunopathological mechanisms, such as enhanced leukocyte arrest and activation in hepatic tissue (our unpublished data, manuscript in preparation). Therefore, the elevation of pro-inflammatory cytokine production and consequent liver injury seen in the absence of IFN-γ appears to account for the worse outcome after DENV infection in mice.

Several studies have demonstrated the IFN-γ-inductive role played by IL-12 and IL-18 during experimental models of viral infections [20], [21], [42]. Here, we have shown that IL-12 and IL-18 were produced early after DENV infection. The kinetics of production of these cytokines was compatible with their inductive role of IFN-γ production. In support of the latter possibility, IL-12p40^−/−^ and IL-18^−/−^ mice presented marked reduction in IFN-γ production after DENV infection. In addition, absence of one of these cytokines led to worsening of dengue disease, despite a small delay in peak of DENV-induced alterations. Of note, only during simultaneous blockade of both IL-12 and IL-18, there was complete abrogation of IFN-γ production. Interestingly, IL-12^−/−^ mice treated with IL-18 bp presented marked enhancement of splenic viral loads already at the 5^th^ day post DENV-2 infection and disease seen in these mice was very similar to that found in infected IFN-γ^−/−^ mice. Thus, IL-12 and IL-18 act synergistically to induce IFN-γ production during DENV infection. Of note, IL-18 production has been shown to be dependent on inflammasome complex activation [43], suggesting that this molecular scaffold may play a role in the control of IFN-γ production and in host resistance to DENV infection.

IL-18 is known to augment IL-12-induced IFN-γ production by T and NK cells [20], [21], [42], [44], and absence of IFN-γ in infected mice is known to abolish both NK cell and CTL responses during viral infections [42], [44]. Our data suggest that, upon infection, NK and NKT cells are the cell populations involved in early IFN-γ production and that CD8^+^ and CD4^+^ T cells are the main IFN-γ producers at later moments of response to infection (7^th^ day). IFN-γ production by CD4^+^ T cells during experimental DENV infection has been previously demonstrated [45]. In addition, CD8 T cell activation has been associated to protection to DENV primary infection in mice [46], [47]. Our data showing a significant increase in IFN-γ^+^ NK and NKT cells and the finding that IFN-γ^−/−^ mice succumb very early to infection suggest a important role for these cell populations in mediating resistance to DENV infection during its initial phases. Of note, NK cell activation early after experimental DENV infection has been previously demonstrated [44]. Interestingly, increased percentages of NK cells and of activated NK cells were also associated with milder DF, whereas reduced cell counts, low percentages and lack of activation markers (comparable to healthy controls) were associated with evolution to DHF in patients [48], [49]. Altogether, these observations suggest that sequential and coordinated IFN-γ production by these lymphocytes populations during DENV infection is an event of extreme importance for host resistance to disease.

However, it remains to be shown the antigenic specificity of these IFN-γ-producing lymphocytes in the studied experimental settings. In addition, whether these cells are poly-functional and secrete other cytokines or present other effector functions remain to be studied. In this regard, it has been demonstrated that development of subclinical secondary infection in school children is associated with increased proportions of DENV-specific TNF-α, IFN-γ and IL-2-producing CD4^+^ and CD8^+^ T cells [50], suggesting that poly-functional responses correlate with protection to severe disease manifestation. On the contrary, cytokine-producing T cells (especially TNF-α and/or IFN-γ) were associated with DHF development in patients and these DHF associated, cytokine-producing T cells were shown to be negative for CD107a staining, suggesting that these lymphocyte populations represent mono-functional or oligo-functional T cells [51]. Therefore, assessment of the pattern of T cell cytokine production and of the mechanisms controlling such polyfunctionality (whether IL-12 and or IL-18 are involved in such control) may provide important information regarding protective *versus* pathogenic responses to DENV infection and may bear relevance during development of vaccinal strategies. At the moment, these subjects have been matter of ongoing analysis in our experimental infection model.

Apart from promotion of NK and CTL responses, IFN-γ seems to be important for viral clearance by induction of NO production. It has been shown that NOS2 expression is increased upon DENV infection in humans and that this expression in peripheral blood monocytes of DF patients was found to correlate with the late acute phase of disease and preceded the clearance of DENV from monocytes [52]. Hence, NO production was associated with less severe form of dengue disease in humans [53]. Here, we demonstrate that NOS2 expression is increased during DENV infection and that this expression is controlled by IFN-γ production, once IFN-γ^−/−^ and IL-12p40^−/−^ mice treated with IL-18 bp presented reduced NOS2 expression. In addition, IFN-γ stimulation was necessary for NO production by DENV-infected DCs, *in vitro*. Importantly, blockade of NOS2 action was associated with enhanced viral loads after infection, and more severe disease manifestation, even in the presence of high levels of IFN-γ. Of note, NO is able to inhibit DENV replication in human cells *in vitro* [54], [55], an effect associated with inhibition of DENV associated polymerase activity [54]–[56]. Thus, NOS2-mediated NO production is pivotal for resistance to DENV infection and this seems to be a major pathway involved in IFN-γ-mediated resistance to disease. However, in the absence of NOS2, animals die with a slower kinetics than IFN-γ^−/−^ mice, suggesting that mechanisms in addition to NOS2-mediated NO production may be relevant for IFN-γ-mediated host protection to infection. This could involve the presence of CTL responses and NK cells, but not NKT cells, which seem to play detrimental role in experimental DENV infection [57]. These IFN-γ-dependent and NOS2-independent mechanisms are currently being investigated in our laboratory.

However, other studies have demonstrated a pathogenic role for NO during DENV infection. Utilizing human cell lines and experimental mouse infection, it has been shown that overproduction of NO could lead to endothelial cell damage, and cross-reactive antibodies against endothelial cells, present during DENV infection, were found to induce cell damage in an NO-dependent manner [58]. For example, Yen and coworkers have found that tissue hemorrhage after experimental DENV infection was dependent upon reactive nitrogen species production by endothelial cells. This event was associated with increased endothelial cell apoptosis during infection [59]. Although NOS2 inhibition resulted in reduced hemorrhage, viral replication was not evaluated. In addition, the increased hemorrhage displayed after NO production seemed to be an endothelial cell-associated phenomenon and was potentiated by TNF-α and reactive oxygen species (ROS). On the contrary, IFN-γ-mediated NO inhibition of viral replication was demonstrated especially in leukocytes population both in human and mouse settings [52]–[56]. Our results showed that NOS2 staining during DENV-2 infection in the present model was mainly associated to leukocytes. These findings suggest that NO may have a dual role during DENV infection and that this is associated with the cell populations involved in NO production and on the presence of additional inflammatory mediators. NO production by infected leukocytes may be associated to control of viral replication and prevention of disease evolution, while NO production by endothelial cells, especially in the presence of TNF-α and ROS, would favor cell death and more severe disease manifestation. Additional experiments evaluating cell-specific NOS2-deficient mice will help in answering the latter hypothesis and aid in defining other roles of NO in the context of experimental dengue.

In conclusion, we have demonstrated that IFN-γ production is essential for host resistance to DENV infection. IFN-γ production upon infection is controlled by concomitant production of IL-12 and IL-18 and the IFN-γ-dependent mechanisms associated to resistance to dengue disease involve NOS2 up-regulation and consequent NO production. In the absence of these molecules, there is enhancement of viral burden and more severe manifestation of dengue disease. Thus, IFN-γ induction helps to orchestrate immune response maturation, control of viral replication and regulation of inflammatory response during host response to DENV infection, defining the outcome of dengue disease. Despite extrapolation of this experimental scenario to human infection requires further investigation, we may suggest that strategies that improve the production of IFN-γ-mediated immunity by the host could be useful during the control of primary infection by Dengue virus.

## Supporting Information

Figure S1 **Gating strategy utilized for analysis and representative histograms of IFN-γ production after DENV-2 infection.** WT mice were inoculated with 10LD_50_ of DENV-2 and at the indicated timepoints, IFN-γ intracellular staining in splenic cells was assessed by FACS analysis utilizing the following gating strategy (A) Lymphocyte/monocyte population was isolated among total events as the region R1. 50,000 events at R1 were collected for analysis (left panel). At this region, the cell population positive for IFN-γ staining defined as total IFN-γ^+^-cells (middle panel - R2). Right panel A contains representative histograms of total IFN-γ^+^-cells in each group analyzed. (B) At region R1 in panel A, CD4^+^ cells were isolated (R3 in left panel B), and the cell population positive for IFN-γ staining among them, defined as CD4^+^ IFN-γ^+^-cells (middle panel B - R4). Right panel B contains representative histograms of CD4^+^ IFN-γ^+^-cells in each group analyzed. (C) At region R1 in panel A, CD8^+^ cells were isolated (R5 in left panel C), and the cell population positive for IFN-γ staining among them, defined as CD8^+^ IFN-γ^+^-cells (middle panel C – R6). Right panel C contains representative histograms of CD8^+^ IFN-γ^+^-cells in each group analyzed. (D) At region R1 in panel A, cells were sorted by their staining for CD3 and NK1.1 (left upper panel D). CD3^+^ NK1.1^+^ cells were isolated (R7 at the upper left panel D), and the cell population positive for IFN-γ staining among them, defined as CD3^+^ NK1.1^+^ IFN-γ^+^-cells (upper middle panel D – R9). Upper right panel D contains representative histograms of CD3^+^ NK1.1^+^ IFN-γ^+^-cells. CD3^−^ NK1.1^+^ cells were isolated (R8 at the upper left panel D), and the cell population positive for IFN-γ staining among them, defined as CD3^−^ NK1.1^+^ IFN-γ^+^-cells (bottom left panel D - R10). Bottom middle panel D contains representative histograms of CD3^−^ NK1.1^+^ IFN-γ^+^-cells. Groups analyzed were Not infected animals (dotted line), animals in the 5^th^ day post infection (dashed lines) and animals at the 7^th^ day post infection (continuous line). Grey filled histograms represent Isotype-stained cells. NI: Not infected. dpi: days post-infection.
(TIF)

Figure S2 **IL-23 does not participate in IFN-γ-mediated resistance to DENV infection.** (A–D) WT and IL-23p19^−/−^ mice were inoculated with 10LD_50_ of DENV-2 and at the seventh day of infection, the following parameters were assessed: IFN-γ concentration in serum (A), measured by ELISA; platelet counts (B) and hematocrit (C) in blood; Viral loads recovered from the spleen, by plaque assay (D). Results are expressed as mean ± SEM (except for D, expressed as median) and are representative of at least two independent experiments. N = 4 mice per group. * P<0.05 *vs.* NI. # P<0.05 *vs.* WT. NI: Not infected. ND: Not detected.
(TIF)
