# Supplementary figures and images for: IFN-γ Production Depends on IL-12 and IL-18 Combined Action and Mediates Host Resistance to Dengue Virus Infection in a Nitric Oxide-Dependent Manner
Source: PLoS Negl Trop Dis. 2011 Dec 20;5(12):e1449. doi: 10.1371/journal.pntd.0001449 (PMC3243710; doi:10.1371/journal.pntd.0001449)

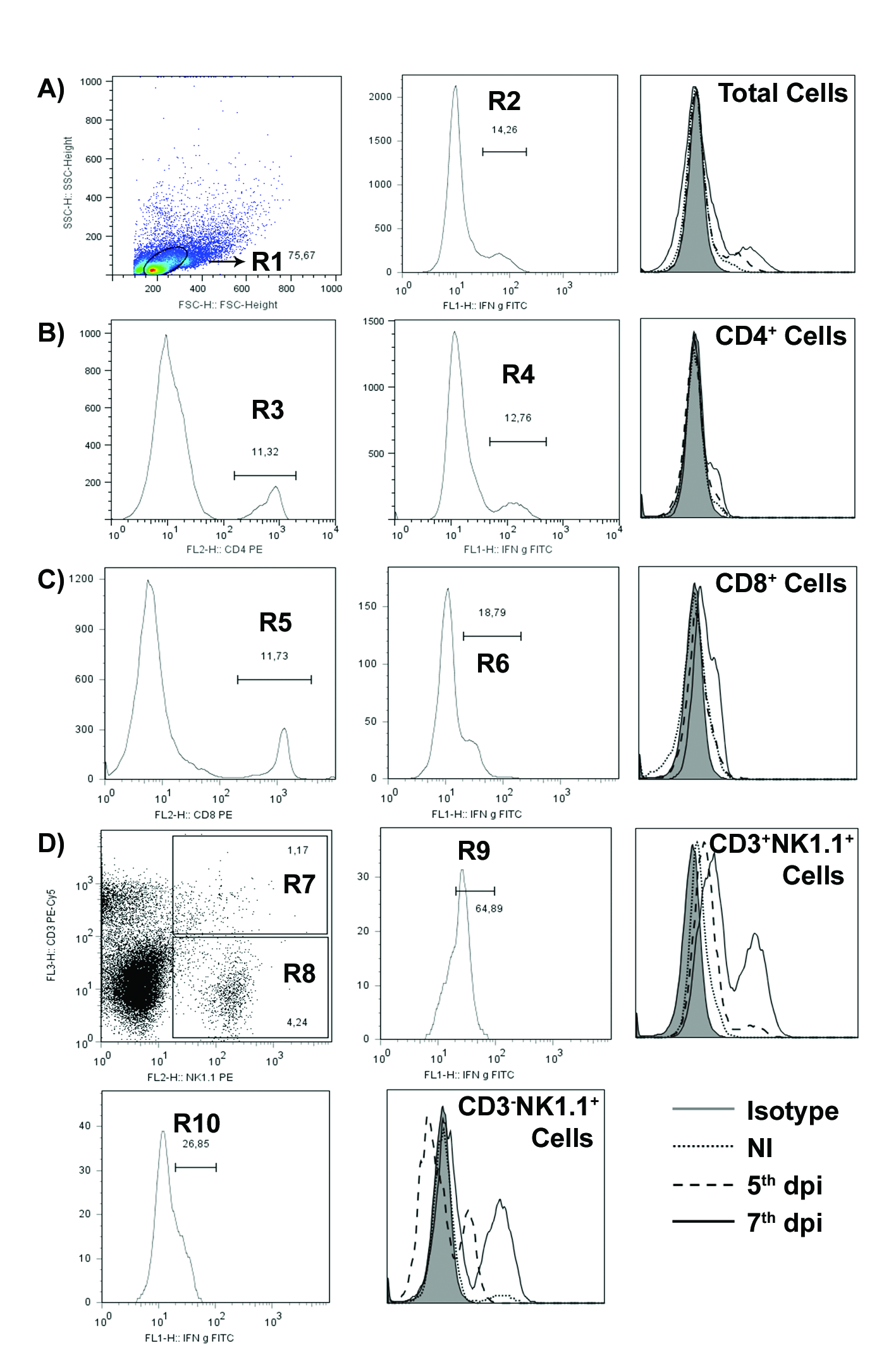

Supplement: Figure S1 — Gating strategy utilized for analysis and representative histograms of IFN-γ production after DENV-2 infection. WT mice were inoculated with 10LD50 of DENV-2 and at the indicated timepoints, IFN-γ intracellular staining in splenic cells was assessed by FACS analysis utilizing the following gating strategy (A) Lymphocyte/monocyte population was isolated among total events as the region R1. 50,000 events at R1 were collected for analysis (left panel). At this region, the cell population positive for IFN-γ staining defined as total IFN-γ+-cells (middle panel - R2). Right panel A contains representative histograms of total IFN-γ+-cells in each group analyzed. (B) At region R1 in panel A, CD4+ cells were isolated (R3 in left panel B), and the cell population positive for IFN-γ staining among them, defined as CD4+ IFN-γ+-cells (middle panel B - R4). Right panel B contains representative histograms of CD4+ IFN-γ+-cells in each group analyzed. (C) At region R1 in panel A, CD8+ cells were isolated (R5 in left panel C), and the cell population positive for IFN-γ staining among them, defined as CD8+ IFN-γ+-cells (middle panel C – R6). Right panel C contains representative histograms of CD8+ IFN-γ+-cells in each group analyzed. (D) At region R1 in panel A, cells were sorted by their staining for CD3 and NK1.1 (left upper panel D). CD3+ NK1.1+ cells were isolated (R7 at the upper left panel D), and the cell population positive for IFN-γ staining among them, defined as CD3+ NK1.1+ IFN-γ+-cells (upper middle panel D – R9). Upper right panel D contains representative histograms of CD3+ NK1.1+ IFN-γ+-cells. CD3− NK1.1+ cells were isolated (R8 at the upper left panel D), and the cell population positive for IFN-γ staining among them, defined as CD3− NK1.1+ IFN-γ+-cells (bottom left panel D - R10). Bottom middle panel D contains representative histograms of CD3− NK1.1+ IFN-γ+-cells. Groups analyzed were Not infected animals (dotted line), animals in the 5th day post infection (dashed [file pntd.0001449.s001.tif]
